# Editorial: Women in neurodegeneration

**DOI:** 10.3389/fnins.2024.1388520

**Published:** 2024-03-18

**Authors:** Sukanya Saha, Marija Cvetanovic

**Affiliations:** ^1^Neurobiology Laboratory, Department of Health and Human Services, National Institute of Environmental Health Sciences, National Institutes of Health, Research Triangle Park, Durham, NC, United States; ^2^Department of Neuroscience, University of Minnesota, Minneapolis, MN, United States

**Keywords:** women, neurodegeneration, aging, oligodendrocyte, sex-differences, cognitive testing

While the gender gap in education seems to be closing, based on the 2017 UNESCO Institute for Statistics's report <30% of researchers worldwide are women. Thus the gender gap in science is still wide and it remains likely that when asked to picture a scientist most people will imagine a man. With solemn acknowledgment that gender equality in science requires more work, this Research Topic celebrates women in science and in particular in the field of neurodegeneration, a field of science very relevant to society. Neurodegeneration is an irreversible and deleterious phenomenon, where neural cells become dysfunctional and eventually die. Depending on the brain region involved, neurodegeneration causes different loss of function, including cognitive and motor decline (Dugger and Dickson, 2017; Erkkinen et al., 2018). The huge upsurge in the prevalence of neurodegenerative diseases has become a burden on our society and healthcare system, already affecting millions worldwide. Thus, over the past few decades, there has been numerous ongoing studies to help advance our understanding of these diseases. Among these are some breakthrough discoveries carried out by women scientists. Aiming to advance the evaluation of women's contributions to Neuroscience, this special edition Research Topic “*Women in neurodegeneration*” highlights and emphasizes the contribution of women to neurodegeneration through showcasing their forward-looking, and significant impact on the field.

Mouse models of neurodegenerative diseases are an essential tool in the field of research on neurodegenerative diseases (Gitler et al., 2017). While the role of sex as a biological variable in these models is increasingly appreciated (Cosgrove et al., 2007), very few studies are sufficiently able to examine both male and female mice. A study led by Tkáč et al. used magnetic resonance spectroscopy at 9.4 tesla magnetic field to investigate neurochemicals in the several brain regions, including brainstem, cerebellum, cortex, hippocampus, hypothalamus, and striatum of both male and female mice at several different ages. Authors observed significant sex and age-dependent differences in the neurochemical levels in most regions except hypothalamus, with males having consistently higher levels compared to females. These findings emphasize the importance of including and matching both sexes when designing experiments. They may also carry the implication that aging and neurodegeneration differently impact male and female brains and that successful future treatments may need to be gender specific.

Aging is a critical risk factor for cognitive impairment and dementia (Hou et al., 2019). Therefore, it is important to characterize the extent, severity and type of cognitive impairments at different stages of disease progression. Holz et al. describe a novel content validity evidence of a direct complex 6-domain functionality test intended for this purpose, the Brief Instrument for Direct Functionality Assessment (BIDFA). BIDFA functionality domains involve both semantic memory and procedural memory and thus this test has good content validity with a wider range of total and subdomain scores. The authors, hence, expect that the neuropsychological BIFDA test would help assess the functional status of older adults efficiently in an abbreviated context without any secondhand interferences.

Not unlike women in science there are brain regions and brain cell types that do not receive enough attention.

Vascular dysfunctions are another important contributor to neurodegenerative diseases. Consequences of cerebral strokes are well-studied. However, while cerebellum contains most of the brain neurons, it is rarely included in stroke studies. As a consequence much less is known about long-term cognitive and motor impairments after cerebellar, rather than after cerebral, strokes. The retrospective study by Ewald et al. investigated neuropsychiatric performance in patients in a chronic phase (1.3 year) following cerebellar ischemic and hemorrhaging stroke. Cerebellar strokes occur more in males and can be fatal in the acute phase. Considering the important role that cerebellum plays not only in movement but also in cognition and affect, the main goal of this manuscript was to investigate the long-term consequences of cerebellar stroke in patients. While in most examined cases, authors found marginally decreased performance in neuropsychiatric tests, some patients suffered from significant cognitive impairments. Thus, this study indicates that impairments caused by cerebellar stroke are in large part reversible for most patients.

Most neurodegenerative studies focus on neuronal dysfunction and loss. While often ignored, it is becoming increasingly evident that glial cells, including astrocytes, microglia and oligodendrocytes play critical roles in the pathogenesis of neurodegenerative diseases. The study by Schuster et al. highlights the importance of oligodendrocyte dysfunction in Spinocerebellar ataxia type 3 (SCA3). SCA3 is an inherited neurodegenerative disease caused by the expansion of CAG repeats in the Ataxin 3 gene. Dr. Hayley McLaughlin's laboratory has previously demonstrated perturbation in oligodendrocyte numbers and maturation in mouse model of SCA3. Here they demonstrate a decrease in oligodendrocyte function by showing reduced myelination in SCA3 patients. Furthermore, the authors found an intriguing spatio-temporal pattern of oligodendrocyte changes in mouse models of Spinocerebellar ataxia type 3 (SCA3). Authors demonstrated that the decrease in oligodendrocyte transcripts in disease affected regions occurs concurrently with the onset of motor deficits. These results expand our understanding of role of oligodendrocytes in the regional vulnerability in neurodegeneration. Another significant outcome of this study is identification of timepoints and target regions for biomarker assessment and therapeutic intervention in SCA3.

We hope that celebrating these important studies conducted by groups of amazing women scientists will increase appreciation and efforts to achieve gender equality in science. And perhaps start picturing scientists as a woman in a lab coat.

## Author contributions

SS: Writing – original draft, Writing – review & editing. MC: Writing – original draft, Writing – review & editing.
